# *Bacillus*-Loaded Biochar as Soil Amendment for Improved Germination of Maize Seeds

**DOI:** 10.3390/plants12051024

**Published:** 2023-02-23

**Authors:** Vanja Vlajkov, Ivana Pajčin, Snežana Vučetić, Stefan Anđelić, Marta Loc, Mila Grahovac, Jovana Grahovac

**Affiliations:** 1 Faculty of Technology Novi Sad, University of Novi Sad, Bulevar Cara Lazara 1, 21000 Novi Sad, Serbia; 2 Faculty of Technical Sciences, University of Novi Sad, Trg Dositeja Obradovića 6, 21000 Novi Sad, Serbia; 3 Faculty of Agriculture, University of Novi Sad, Trg Dositeja Obradovića 8, 21000 Novi Sad, Serbia

**Keywords:** plant growth promotion, biocontrol, enzymatic activity, IAA, surfactin, ACC deaminase, root length, shoot length, seedling vigour index

## Abstract

Biochar is considered one of the most promising long-term solutions for soil quality improvement, representing an ideal environment for microorganisms’ immobilization. Hence there is a possibility to design microbial products formulated using biochar as a solid carrier. The present study was aimed at development and characterization of *Bacillus*-loaded biochar to be applied as a soil amendment. The producing microorganism *Bacillus* sp. BioSol021 was evaluated in terms of plant growth promotion traits, indicating significant potential for production of hydrolytic enzymes, indole acetic acid (IAA) and surfactin and positive tests for ammonia and 1-aminocyclopropane-1-carboxylic acid (ACC) deaminase production. Soybean biochar was characterised in terms of physicochemical properties to evaluate its suitability for agricultural applications. The experimental plan for *Bacillus* sp. BioSol021 immobilisation to biochar included variation of biochar concentration in cultivation broth and adhesion time, while the soil amendment effectiveness was evaluated during maize germination. The best results in terms of maize seed germination and seedling growth promotion were achieved by applying 5% of biochar during the 48 h immobilisation procedure. Germination percentage, root and shoot length and seed vigour index were significantly improved when using *Bacillus*-biochar soil amendment compared to separate treatments including biochar and *Bacillus* sp. BioSol021 cultivation broth. The results indicated the synergistic effect of producing microorganism and biochar on maize seed germination and seedling growth promotion, pointing out the promising potential of this proposed multi-beneficial solution for application in agricultural practices.

## 1. Introduction

The projections of The Food and Agriculture Organization of the United Nations (FAO) confirm the expected rise in the world’s population by 2050 to 9.1 billion. To meet the need in terms of global demands on crop yield, the agricultural system depends on the development of solutions beneficial from the perspective of efficient plant cultivation, and on the other hand, minimizing the ecological footprint. Green biotechnological products are the main focus of researchers and agricultural producers as promising solutions meeting both of the abovementioned requirements. The plant growth-promoting (PGP) bacteria are recognised as key game changers in the development of eco-friendly microbial products intended for use in agriculture [1,2,3,4]. The beneficial activities of the PGP strains are reflected through multiple mechanisms of action, characterised as direct or indirect, including nitrogen fixation, nutrient solubilization, phytohormones, hydrolytic enzymes, antibiotics and siderophores synthesis and induction of systematic resistance of plants [5,6,7]. Taking into account all the beneficial activities, products based on PGP strains could be used as biofertilizers, biocontrol agents, phyto-stimulators and soil quality boosters. Previous studies confirmed the ability of PGP-based products to contribute to plant growth evaluated through the observed increase in shoot and root length, biomass, seed germination and leaf size [5]. The members of the genus *Bacillus* are one of the most studied representatives of PGP rhizobacteria [8]. The high potential of *Bacillus* species lies in their ability to produce a number of beneficial components including IAA (indole acetic acid), hydrocyanic acid (HCN), siderophore, hydrolytic enzymes, compounds with antimicrobial activity, as well as their abilities of phosphate solubilisation and nitrogen fixation [9]. Additional advantage comes from the fact they are spore-forming bacteria. Compared to vegetative forms, spores are more robust and resistant, which makes them more convenient for manipulation in the production process, simultaneously providing a longer shelf-life for the final product [10].

The scientific work of many research groups is focused on collecting data on beneficial microorganisms that represent a significant potential for application in agricultural practice [11]. On the other hand, the number of commercial products available on the market does not follow this trend. Many products do not achieve satisfactory efficiency in the laboratory, under controlled conditions (greenhouse) and in the field, and the reason for this is explained by inadequate approaches to the formulation of final products. The role of formulation in the biotechnological production of microbial-based products lies in designing a suitable microenvironment for active components, i.e., cells of microorganisms, including their physical or chemical protection over time. The essential function includes favouring the activity of the product and the competitiveness of microorganisms compared to other natural strains better adapted to the conditions of the application site [12,13,14].

The characteristics of the selected producing microorganisms strictly dictate the potential approaches in the formulation of the final product. Due to their specific properties, the representatives of the *Bacillus* genus are defined as more suitable candidates compared to a large number of competing strains. This primarily refers to the ability to produce endospores, which are characterised by exceptional stability in extreme environmental conditions, thus also the possibility of applying chemical and physical formulation techniques that would be unacceptable in the case of unstable forms of microorganisms [12].

Possible types of formulation are divided into two major groups, liquid and solid [13,14]. The most common solid formulations of the product include powdery consistency, in the form of micro granules and wettable powder. Liquid forms of the product include emulsions, oil dispersions, concentrate suspensions and water-soluble granules [15,16]. A special group of products includes cell-free formulations, i.e., filtrate or supernatant of cultivation liquids. In the field of technology development of solid product forms, increasing interest is attracted by cell immobilisation techniques using polysaccharides, as well as products obtained by performing solid-state cultivation using agro-industrial waste. This type of production opens up the possibility of applying co-cultivation of several beneficial strains, as well as the possibility of applying solid substrates independently or in combination with liquid waste materials [13].

Since the formulation of biocontrol agents is a critical factor regarding their commercial use, the basic requirements that must be met, above all, relate to ensuring cell viability and a high concentration of active components per product unit. On the other hand, the economic aspect of the formulation procedure is also mentioned as an important factor [13]. Additional challenges from the point of view of commercialization relate to the shelf life of microbial-based products and defining the formulation procedure that will ensure the stability of the product for as long as possible [14,17]. The shelf life of microbial-based products including spores is on average 1–3 years [10]. The strict requirements regarding the storage and transport conditions for biological products for plant protection are often recognized as limiting factors in their production development. A potential solution to overcome the mentioned limitations is found in the application of biocompatible carriers that are included as components of the final product [18].

Biochar is a solid material usually produced by pyrolysis, hydrothermal carbonization, gasification, torrefaction or flash carbonization of biomass, mostly plant residues [19]. The importance of biochar from the point of view of agricultural production lies in the fact that it is considered the most promising long-term solution for the improvement of soil quality [20,21]. On the other hand, the typical structure of biochar, which implies the existence of a wide distribution of pore sizes, represents an ideal environment for microorganisms’ attachment and immobilisation. Biochar is characterised by high water retention capacity, oxygen availability, and also the role of a carbon and mineral source, which enables the longer-term survival of bacteria [22]. The immobilisation of bacteria of the genus *Bacillus* on biochar particles showed a positive effect both on the quality of the soil itself and on the yield of cultivated crops. It is about the synergistic activity of bacteria characterised as plant growth promoters and the beneficial effects manifested by the presence of biochar in the soil, in terms of its regeneration and improvement of physical properties, structure, ability to retain nutrients and general productivity [21,23].

The importance of the integrated application of biochar and plant growth-promoting bacteria (PGP), among which *Bacillus* species take the leading place, is reflected in the additional potential of products formulated in this way as ecological substitutes for conventionally applied agents for improving soil quality. The multiple mechanism of action originating from microbial activity, i.e., living cells of beneficial microorganisms in terms of nutrient solubilisation, production of plant hormones, siderophores, exopolysaccharides and phytoremediation of pollutants, coupled with all aspects of the positive impact of biochar results in an overall improvement in many aspects of agricultural production [18,23,24]. The centre of research focused on the impact of these kinds of products on soil quality and plant growth were also the problem of cultivating corn in conditions of limited water availability. The characteristic of corn production is the high nutritional requirements compared to other crops and the problem of their insufficiency in the soil due to intensive consumption. The results of research by other authors confirmed the potential of the combined application of useful strains of microorganisms and biochar, contributing both to the improvement of soil quality as the basic resource of production, and to the yield and quality of the obtained crops [25].

The incorporation of beneficial microorganisms into a solid carrier, results in a high concentration of active components per unit mass of the final product, which simultaneously implies a high efficiency of the product at the application site. Taking into account their specific structure, biochar particles play a protective role for biological agents, achieving additional stimulation of their beneficial action, but also high viability, which is crucial in terms of the possibility of commercialization of the final product [24,25,26,27].

According to the summarised literature data, the hypothesis of this study was that soybean biochar could be successfully applied as a solid carrier for *Bacillus* PGP strain, in order to develop microbial soil amendment with both beneficial effects on soil quality and plant growth and development. The formulation method, including the biocompatible carriers is recognised as a promising tool in sustainable agriculture, providing a protective role for the producing microorganism as the active component of the product, but also contributing to the overall positive impact on plant cultivation. Maize has been chosen as the economically most important crop worldwide, with an estimated global annual production of 1137 million tons and 197 million hectares under this crop around the world [28]. The principal aim of this study was to investigate conditions for immobilisation of *Bacillus* sp. BioSol021, with proven PGP activities, to soybean biochar in order to obtain soil amendment with maximised activity in terms of maize seed germination and initial seedling development. 

## 2. Results and Discussion

### 2.1. Biochar Characterization

**Figure 1:**
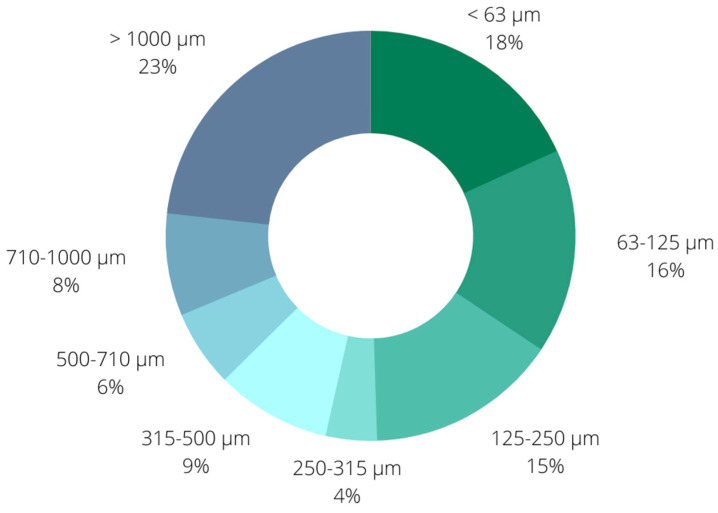
Particle size distribution of biochar particles.

The initial step in biochar characterization was examination of particle size distribution. This was achieved by sieving with the ISO 3310-1:2016 standard set of sieves (aperture sizes of 63, 125, 250, 315, 500, 710, and 1000 µm) (Figure 1). As can be seen, the dominant fraction were particles larger than 1000 μm with a share of 23%, followed by the smallest fraction including particles smaller than 63 μm (18%) and the fraction with biochar particles in the size range 63–125 μm. The presented results also point out the dominant presence of particles smaller than 250 μm (49%), while the share of particles larger than 500 μm was 37%. 

**Figure 2:**
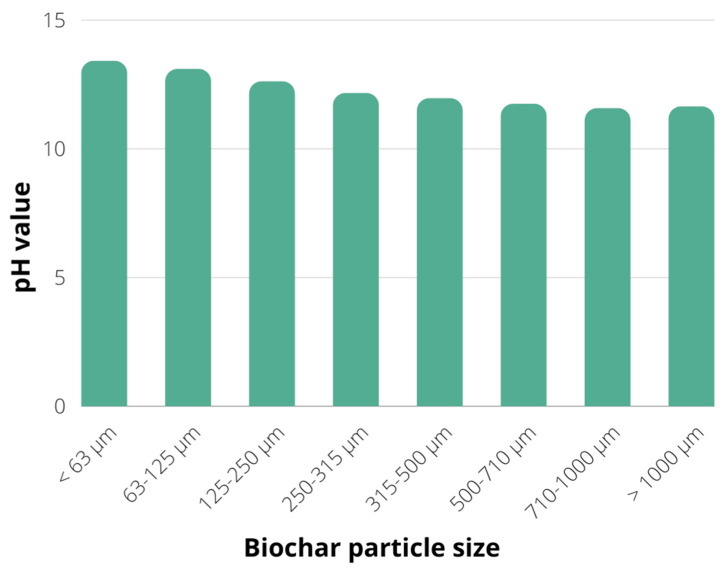
pH value of different fractions of biochar particles.

The next step was to determine the pH value of each biochar particle fraction, as described in Section 3.1. The decrease in pH value could be observed with the increase in particle size, while all fractions have shown alkaline pH values in the range of 11.64–13.41 (Figure 2). The alkaline pH value of a biochar suspension results from the alkali salts remaining in biochar, while the organic substances are eliminated from the feedstock organic matrix during the pyrolysis process [29]. Hence the biochars produced by pyrolysis usually have a pH value over 7, with an observed increase in pH value following the increase in the pyrolysis temperature [30,31]. This hypothesis is also confirmed for soybean stover biochar, with a pH value of 7.27 ± 0.03 for biochar obtained by pyrolysis at 300 °C, while the biochar produced at pyrolysis temperature of 700 °C has shown a pH value of 11.32 ± 0.02, mostly due to the feedstock dehydration and removal of acidic groups at higher temperatures resulting in more alkaline biochar surface [32]. Biochar’s alkaline nature provides the release of alkaline substances in the soil, counteracting soil acidification [33]. Furthermore, the higher pH value of biochar promotes its application as a soil conditioner to improve soil health, mostly due to improved soil aeration, water content and redox potential, as well as due to reduction in heavy metal contamination by electrostatic interactions [34,35]. 

**Figure 3:**
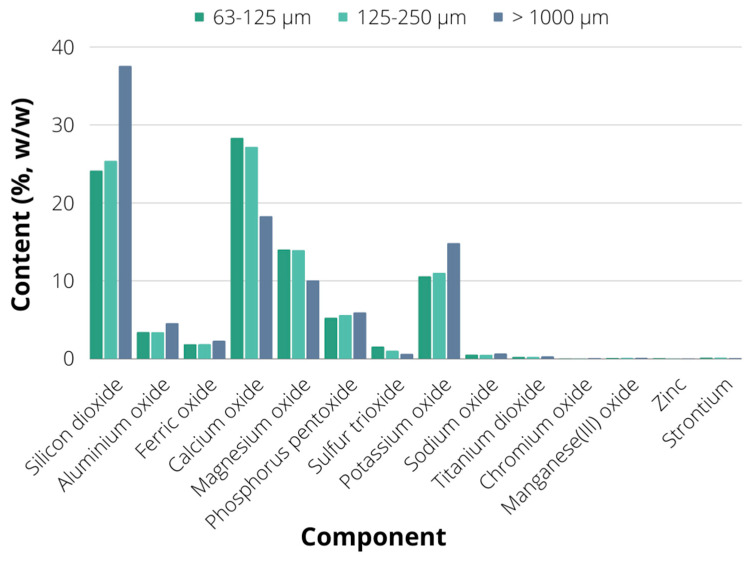
The results of X-ray fluorescence spectroscopy (XRF) analysis of biochar.

In order to better understand the chemical composition of soybean biochar, XRF (X-ray Fluorescence Spectroscopy) analysis was employed. The results of XRF analysis for the dominant biochar fractions of particles larger than 1000 μm and sized in ranges of 63–125 μm and 125–250 μm are given in Figure 3. As can be seen, the dominant component of the fraction containing particles larger than 1000 μm was silicon (37.526%), followed by calcium (18.290%), potassium (14.812%), magnesium (10.04%) and phosphorus (5.928%). The high silicon content could be observed for biochar arising from plants with hard protective shells, such as rice husk biochar [31,36]. It was also observed in biochar produced from a mixture of different agricultural waste, including date leaves, ornamental plant waste, grass clippings and coconut leaves, where the silicon dioxide content was increased with the increasing pyrolysis temperature [37]. The crop productivity under stress is enhanced by the silicon presence as it contributes to nutrients availability through chemical dynamics with other soil elements, as well as through the decrease in availability of toxic chemicals in both plants and soil [38,39]. Similar elemental content could be observed in the fraction containing particles whose size was in the range of 125–250 μm, which consisted predominantly of calcium oxide (27.156%), followed by silicon dioxide (25.383%), magnesium oxide (13.911%), potassium oxide (11.013%) and phosphorus pentoxide (5.587%). 

Calcium is a plant essential nutrient known for its dual function: structural, as a component of cell walls and membranes, and signalling role as a second messenger in plant physiological and developmental processes, as well as metabolic processes included in abiotic and biotic stress responses [40]. Magnesium is a component of chlorophyll pigments, hence making it an essential plant nutrient for photosynthetic CO_2_ assimilation [41]. It is also a cofactor of many plant enzymes, thus participating in energy metabolism, nitrogen uptake and utilization, sucrose transport, pollen synthesis, plant-microbe interactions etc. [42,43]. The XRF analysis of the third examined fraction (63–125 μm) has shown similar results to the previously described fraction, with calcium oxide content of 28.311%, silicon dioxide content of 24.125%, magnesium oxide content of 13.987%, potassium oxide content of 10.573% and phosphorus pentoxide content of 5.256%. Considering that these particle fractions were the most represented in biochar (54%), it suggests suitable biochar composition regarding basic plant requirements for nutrients including potassium, phosphorus, magnesium, calcium and silicon. Potassium, calcium, and magnesium are water soluble, while biochar can also release phosphorus [39], thus soybean biochar could be applied as a soil amendment for enhancement of soil fertility through improved macronutrient availability. These elements (K, Ca, Mg and P) are not lost during the pyrolysis process and remain in the biochar due to the low volatility of their oxides, which are formed during the pyrolysis [44] and affect the increase in biochar pH value, making it a more suitable soil amendment, especially for acidic soil remediation [45]. Hence the XRF-observed chemical biochar composition is in accordance with previously discussed results for soybean biochar pH values. 

Furthermore, a significant content of aluminium oxide, ferric oxide, sulphur trioxide and sodium oxide could be found in each examined fraction, suggesting a suitable soil enrichment basis when it comes to nutrients required for diverse plant functions. Sodium is usually considered a “non-essential” and “functional” plant element, especially considering its beneficial plant effects during potassium deficiency due to Na^+^ and K^+^ chemical and structural similarity [46]. Sulphur is usually designated as the 4th macroelement, besides N, P and K, due to its highly important role in plant metabolism, mostly due to inclusion in protein disulphide bonds, amino acids, vitamins, and cofactors [47]. Furthermore, sulphur is contained in several plant secondary metabolites and glutathione, which provides protection against oxidative stresses [48]. Plants require sulphur for many metabolic reactions, photosynthesis, energy metabolism and signalling [49]. Aluminium shows several plant-beneficial functions, such as stimulated root and shoot growth [50], which is usually a consequence of Al-stimulated and increased macronutrient uptake [51]. It also contributes to prevention of abiotic (e.g., ion toxicity and nutrient deficiency) and biotic stresses (herbivores and pathogens) [52], together with beneficial effects on plant metabolism in terms of increased photosynthesis, antioxidant defence, synthesis of proteins and plant growth regulators, as well as improved regulation of carbon and nitrogen metabolism [53]. Iron is the essential plant trace element responsible for several plant metabolic functions, including respiratory electron transport, photosynthesis and cell wall metabolism, with additional beneficial functions in terms of protection against oxidative stress [54,55]. 

The representation of heavy metals in each biochar fraction was very low (less than 0.02%), suggesting non-toxic biochar effects on plants and soil microbiome. According to the EBC (European Biochar Certificate) guidelines [56], the heavy metals of interest detected in soybean biochar were Cd and Zn. Zn content in each fraction was above the requested EBC limit for biochar application in organic and conventional agriculture (200 g/t), while for the Cr content, the fractions of particles with size in range 63–125 μm and 125–250 μm have shown lower content of Cr than the requested limit for organic agriculture (70 g/t), while the Cr content in a fraction of particles larger than 1000 μm was slightly over the limit for conventional agriculture (90 g/t). However, considering the minor abundance of the aforementioned fraction in the bulk material compared to the other two analysed fractions, the soybean biochar could be successfully applied as soil amendment material in both conventional and organic agriculture. These results also point out the necessity of upstream raw material preparation, especially when it comes to mechanical treatment used for obtaining smaller raw material particles, as well as when it comes to biochar production technology and parameters, emphasizing the necessity of pre-treatment and biochar production process optimization in order to obtain suitable biochar composition in terms of particle size and chemical composition [57]. Furthermore, soybean stover biochar has shown remarkable bioremediation capability when it comes to the adsorption of heavy metals and organic contaminants, with 90% removal efficiency of Pb [58], as well as 70% trichloroethylene removal efficiency [32]. 

**Figure 4:**
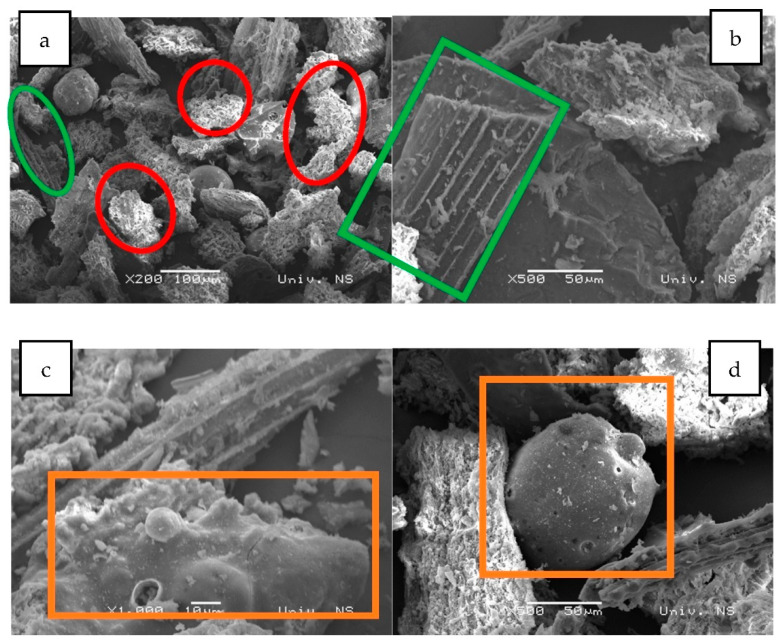
SEM images of biochar particle fraction in the size range 63–125 μm: (**a**)—×200 magnification, (**b**)—×500 magnification, (**c**)—×1000 magnification, (**d**)—×500 magnification.

**Figure 5:**
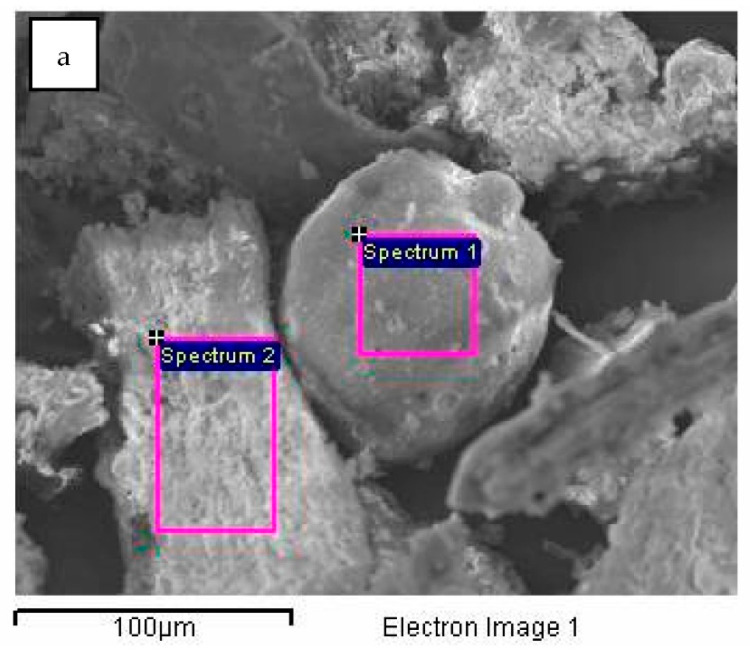

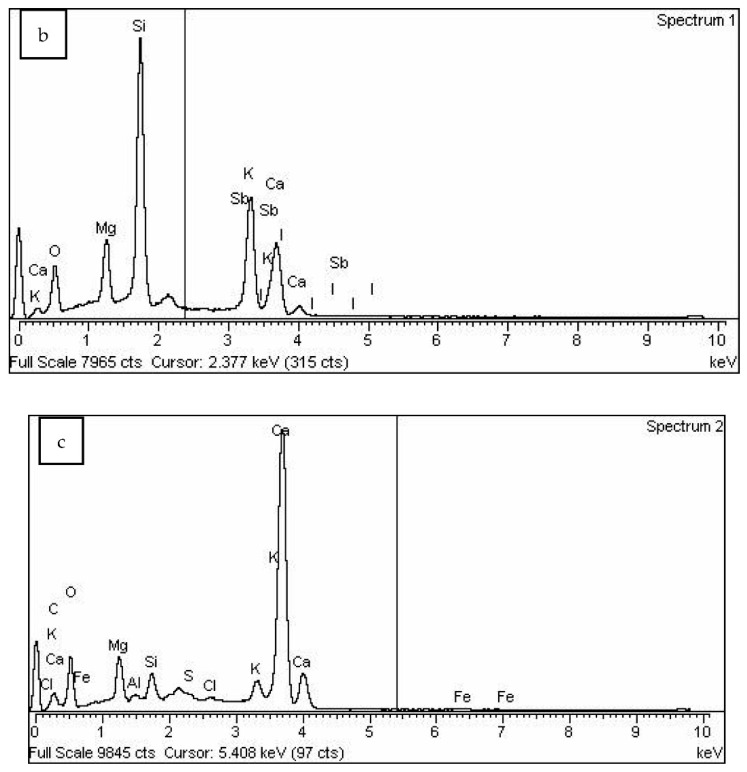
SEM/EDS analysis of biochar particle fraction in the size range 63–125 μm: (**a**)—SEM image, (**b**)—spectrum 1, (**c**)—spectrum 2.

The results of the SEM-EDS (Scanning Electron Microscopy with Energy Dispersive Spectroscopy) analysis of the biochar fraction of particles with sizes in the range 63–125 μm are given in Figure 4 and Figure 5 and Table 1. There could be observed a dominant presence of particles with sharp edges (Figure 4a, red), while the forms arising from the incineration of plant remains are observed to a lesser extent (Figure 4a,b, green). Furthermore, round-shaped particles emerging as a melting consequence (characteristic for thermal treatment) could be observed (Figure 4c,d, orange). EDS analysis has shown a high content of silicon (23.2%), potassium (14.29%), calcium (10.05%) and oxygen (42.62%) for round-shaped particles (spectrum 1), as well as high content of calcium (30.21%), carbon (10.43%) and oxygen (49.30%) for particles with sharp edges (spectrum 2). Particles arising from the carbonization of plant residues have shown higher carbon content (Figure 5 and Table 1—spectrum 2). 

**Figure 6:**
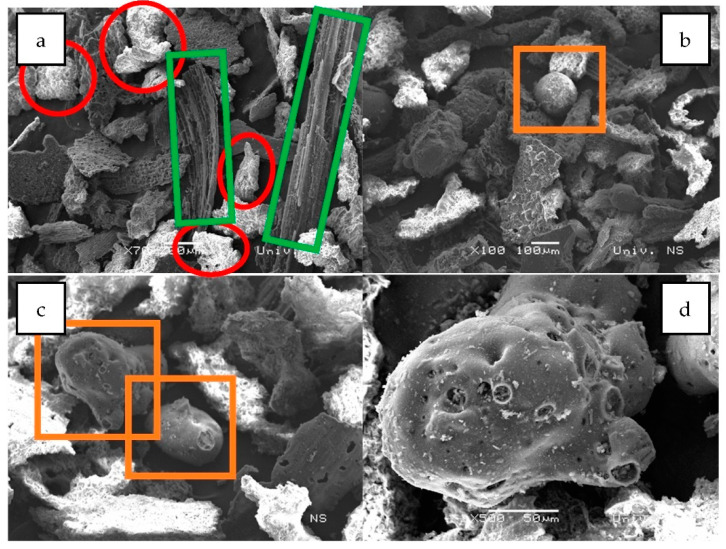
SEM imaging of biochar particles fraction in size range 125–250 μm: (**a**)—×700 magnification, (**b**)—×100 magnification, (**c**)—×100 magnification, (**d**)—×500 magnification.

**Figure 7:**
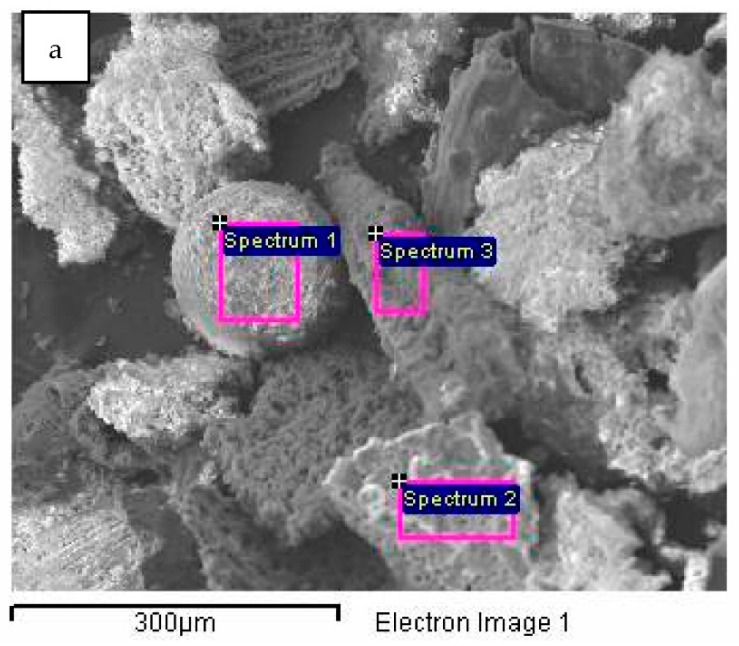

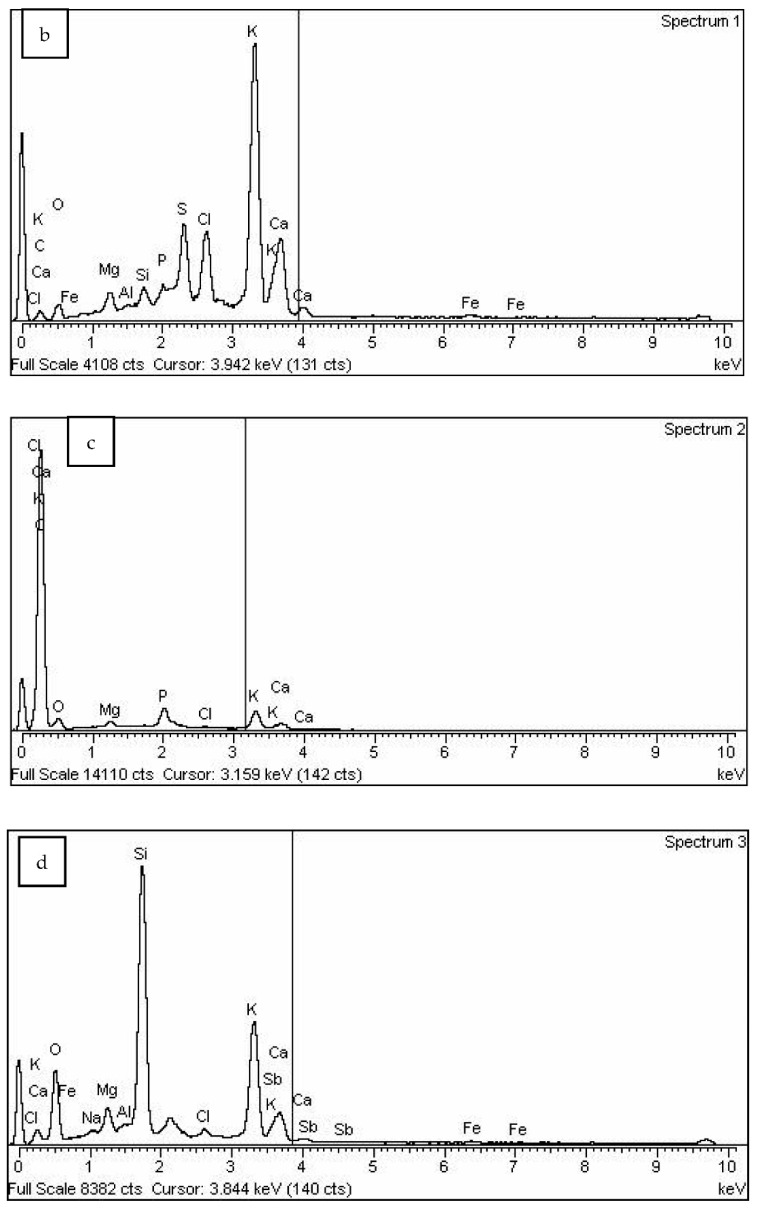
SEM/EDS analysis of biochar particle fraction in size range 125–250 μm: (**a**)—SEM image, (**b**)—spectrum 1, (**c**)—spectrum 2, (**d**)—spectrum 3.

Figure 6 shows the results of the SEM analysis for the biochar fraction of particles with size in the range 125–250 μm. The abundance of round-shaped particles is also low in this fraction (Figure 6a–c, orange), with the dominant presence of particles with sharp edges (Figure 6a, red) and particles arising from carbonization of plant residues (Figure 6a, green). Figure 6d shows an enlarged round-shaped particle with pores with diameters of approximately 10 μm under 500× magnification. As observed previously for soybean stover biochar, higher pyrolysis temperature results in increased biochar surface area, including increased porosity, reduced pore size and appearance of internal pores, mostly due to removal or escape of volatile substances at higher temperatures [32]. The increased porosity due to escape of volatile compounds during pyrolysis improves biochar water retention capacity [59]. The SEM-EDS analysis (Figure 7 and Table 2) has shown similar results to the previous case, with the exception of chlorine’s (7.73%) presence in round-shaped particles (spectrum 1).

**Figure 8:**
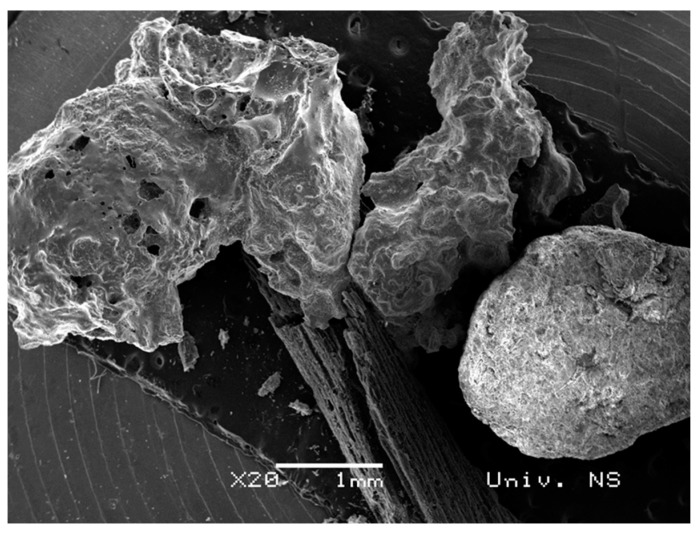
SEM image of biochar for the fraction of particles larger than 1000 μm, ×20 magnification.

The results of the SEM analysis for the biochar fraction of particles larger than 1000 μm are given in Figure 8. This fraction contains particles with highly heterogeneous morphology, but the three most abundant forms (round-shaped particles, particles with sharp edges and carbonized biomass) could also be observed as in the previous cases. Bearing in mind the presented morphology of particles larger than 1000 μm and slightly increased Cr content (XRF analysis), this fraction was no longer used in further experiments. 

### 2.2. Composition of the Bacillus sp. BioSol021 Cultivation Broth

Prior to further investigative steps, the cultivation broth obtained after 96 h cultivation, performed in a 16 L laboratory bioreactor using the selected producing microorganism *Bacillus* sp. BioSol021 under defined experimental conditions, was characterised in terms of crucial parameters including residual cellulose content (2.8 g/L), residual total nitrogen content (0.27 g/L), biomass content 8.47 (log CFU/mL) and pH value (6.3) (Table 3).

### 2.3. PGP and Biocontrol Traits of the Bacillus BioSol021 Cultivation Broth

In order to better understand the possible application of beneficial microorganisms immobilised on biochar as soil amendment agents, cultivation broth of the plant-beneficial strain *Bacillus* sp. BioSol021 was produced to maximise the viable cell number and provide the optimal basis for bacterial immobilisation. Furthermore, PGP and biocontrol traits of the obtained cultivation broth were tested in order to understand the potential of the microorganisms to be immobilised using biochar for agricultural applications. The following PGP/biocontrol traits of the *Bacillus* sp. BioSol021 cultivation broth were tested: surfactin production, IAA production, enzymatic activity of the hydrolytic enzymes (cellulase, xylanase, pectinase and protease), phosphate solubilisation, ammonia production and ACC (1-aminocyclopropane-1-carboxylic acid) deaminase production. The results of the PGP/biocontrol traits’ screening are given in Table 4.

In the present study, the colorimetric method commonly used for rapid and cost-effective surfactin determination described by Yang et al. [60] was employed. The obtained surfactin concentration in the present study using a natural strain originating from the rhizosphere soil and using a cellulose-based medium was 1475 mg/L. Taking into account the extracellular nature of surfactin as a secondary metabolite, the analysis was performed using a cell-free supernatant. Surfactin production is directly influenced by cultivation conditions, and it is described as one of the key perspectives reflecting the optimisation of its biotechnological production [61]. Biosurfactants, including surfactin, have attracted special attention due to their properties, including eco-friendly characteristics, biodegradability and low toxicity. They have been defined as green products thanks to their important role in agriculture and potential for creating a cleaner environment [62]. It was found that surfactin contributes to the improved cellulase activity by reducing the non-productive enzyme binding to lignin and by promoting cellulase binding to cellulose fibres [63], which is of utmost importance for agricultural waste degradation by microbial enzymatic activity, especially in terms of soil quality improvement and soil microbiome diversity increase by providing usable nutrients for soil microflora. 

Furthermore, cellulose has been shown to be a suitable substrate for the induction of surfactin production, as higher levels of gene expression related to surfactin production were found when exchanging glucose for cellulose in the cultivation medium [64]. Lignocellulosic materials are described as one of the most promising renewable feedstocks available in nature, recognised as crucial to the development of a sustainable global economy [65]. Previous studies also addressed the possibility of using agro-cellulose material in medium preparation, as an alternative approach to overcoming the biggest challenge to surfactin’s wider commercialization, which is high production costs. The possibility to substitute, partially, or ideally totally, the expensive synthetic media for agro-industrial residues can significantly contribute to the competitiveness of the developed biotechnological solution. Hemicellulosic corncob liquor was used as an alternative carbon source for *Bacillus subtilis* ICF-PC cultivation and the production of two biosurfactants B-STRR and B-EI24, characterised by a remarkable emulsifying capability, which qualified them for commercial application as bioremediation agents [66]. 

The importance of surfactin in agricultural applications lies in surface activity contributing to the inhibition of biofilm formation thus providing a higher level of plant protection against pathogen colonization, together with induced systemic resistance by induction of defence enzyme synthesis, as shown in maize [67]. The ability to prevent biofilm formation is often related to surfactin’s ability to induce bacterial aggregation, usually as a result of cell wall disruption and peptidoglycan cleavage [67]. Surfactin-induced systemic resistance in plants was also reported previously [68]. The biocontrol activity of surfactin against bacterial and fungal plant pathogens is mostly based on its interaction with lipidic bilayer membranes, where it chelates cations, solubilizes membrane and leads to the formation of pores resulting in cell lysis of pathogens [69]. Its application compared to conventional pesticide agents, results in a lower probability of resistance development, considering its ability to infiltrate and damage fungal cell membranes [70]. Furthermore, surfactin was found responsible for preventing *Fusarium moniliforme* contamination on maize seed kernels, which resulted in improved seed germination and prevention of mycotoxicosis in animals [71]. A number of studies addressed different perspectives on surfactin production aimed at detection of the potential producers and possibilities for further production optimisation. Screening of biosurfactant-producing capability was performed among the 290 *Bacillus subtilis* isolates, out of which 185 isolates showed positive results. Further steps included genetic characterisation, narrowing the selection to the 14 strains with the presence of the specific *spf* gene, and HPLC-MS analysis indicating the most potent producer, with surfactin concentration of 1610 mg/L [72]. Genetic manipulation is also mentioned as a possible approach aimed at increasing the surfactin yield. However, in the study by Geissler et al., the reference strain showed higher surfactin production capacity in comparison with the genome reduced strain with the superior specific growth rate, with the final surfactin concentration 1147.03 mg/L [73].

Indole acetic acid (IAA) is the most common plant hormone belonging to the auxin class, recognised as a potent signalling compound crucial in plant-microbe interactions and involved in key aspects of plant growth and development [74]. IAA promotes the production of longer roots with an increased number of root hairs and root laterals, important from the point of view of plants’ nutrient uptake, consequently leading to higher crop yields. The additional beneficial activity of IAA lies in the stimulation of cell elongation by modifying certain conditions, including an increase in osmotic content and water permeability, and a decrease in wall pressure and cell wall expansion [75,76]. Taking into account the incontestable contribution of IAA to plant growth, a common criterion considered in plant-beneficial bacteria screening includes the ability of investigated strains to synthesize IAA. Representatives of the *Bacillus* genus have previously been proven to be effective in growth and germination of certain plants of economic importance, employing IAA tryptophan-dependent or independent synthesis pathways [77]. In terms of IAA production, the beneficial strains showed a wide range of concentrations, from around 2 ug/mL to values higher than 50 ug/mL [78,79]. In the present study, the obtained concentration of IAA was 15 ug/mL, demonstrating the significant potential of the selected producing strain *Bacillus* sp. BioSol021 for the production of this important plant hormone. Previous studies also investigated the influence of medium composition on IAA production capacity, with the emphasis on carbon source [79]. In the present study, the cellulose-based medium was used for cultivation, indicating the possibility of further development of the bioprocess solution in the direction of utilization of agro-cellulose waste as a suitable low-cost substrate. 

Screening for hydrolytic enzyme activity among *Bacillus* isolates is a common step in the evaluation of the PGP traits, considering that activity of hydrolytic enzymes contribute to better utilization of the nutrients available in the soil by plants and other beneficial soil microorganisms, including improved degradation of harvest residues, and direct antagonism against phytopathogens as a result of cell wall degradation mediated by hydrolytic enzymes. Considering the possible origin and composition of the harvest residues present in the soil, cellulase, xylanase, pectinase and protease activity are of the utmost importance. Cellulases are recognised as the most important class of lignocellulolytic enzymes which are essential for the hydrolysis of agricultural residues due to complete cellulose hydrolysis through consecutive actions resulting in the generation of glucose monomers [80]. Pectin is a component of biomass waste, dominantly present in vegetables and fruits. Sugar beet pulp, apple pomace and citrus waste are defined as examples of biomass high in pectin content, with about 12% to 35% pectin by dry weight. On the other hand, grass and woody biomass aren’t regarded as sources rich in pectin content, with values of 2–10% and 5%, respectively [81]. The pectinolytic activity of enzymes is enabled through depolymerisation and de-esterification reactions resulting in pectin decomposition. Furthermore, pectin is also shown to be a strong surfactin synthesis stimulator, especially during the rhizosphere cross-talk between the plant roots and *Bacillus* PGP bacteria mediated by plant root exudates [82], indicating the necessity for pectinase production in order to metabolize pectin as a biopolymer. Production of proteases is considered one of the major antagonistic traits of PGP bacteria considering their role in phytopathogens’ cell wall degradation [83]. Since plant cell walls contain significant amounts of proteins including xyloglucan endotransglycosidases, extensins and arabinogalactan-rich glycoproteins, efficient biomass decomposition requires activity of proteases as well [84]. As the second most abundant polysaccharide, right after cellulose, xylan covers 33% of total lignocellulosic biomass on a global level. It commonly accounts for 15–30% of hardwoods and around 7–10% of softwoods. Decomposition of xylan is another important step in degradation of agricultural residues and it is possible by the xylanase activity that enables depolymerisation of xylan into simple monosaccharides and xylooligosaccharides [85]. The methods commonly used for the evaluation of enzymatic activity level include determination of halo zones and *EAI*, indicating the ratio of the enzymatic activity related directly to the extracellular enzyme production. The pectinase activity of *Bacillus* representatives measured as hydrolysis zone diameter is commonly in the range of 5–30 mm, divided into groups as poor producers < 5, weak producers > 5, good producers > 10, very good producers > 15, while enzymatic activity index values are between 2.0–5.0 [86,87,88]. In previous studies, the cellulase activity of *Bacillus* genus members are characterised by halo zones in the range of 10–40 mm and *EAI* in the range of 0.2–2.5 [89,90,91]. When it comes to the proteolytic enzymatic activity, most *Bacillus* protease producers provided halo zones between 20–50 mm and *EAI* up to 2.5 [92,93,94,95]. The activity of xylanases produced by *Bacillus* spp. commonly observed in previous studies includes clear zone diameters up to 8 mm [96,97]. In the present study clear zone diameters obtained as a result of cellulase, xylanase, pectinase and protease activity were 54.50 ± 0.50, 32.00 ± 0.50, 21.00 ± 1.00 and 31.00 ± 0.00 mm, with *EAI* values 2.71, 2.85, 3.58 and 1.55, respectively. The results undoubtedly indicated a high potential of producing microorganism *Bacillus* sp. BioSol021, for the production of key hydrolytic enzymes important from the point of view of beneficial activity regarding plant growth promotion and overall improvement of crop cultivation.

Phosphate solubilisation is an important PGP trait considering its necessity for natural phosphorus cycling. Phosphorus is one of the plant essential elements included in different metabolic processes, from photosynthesis and respiration to energy storage [98]. The majority of phosphorus in the soil (over 95%) is present in insoluble forms [99], which cannot be used by plants, combined with its restricted mobility and slow soil diffusion [100], result in the most important limitations for phosphorus uptake by plants. Phosphate-solubilizing bacteria solubilize precipitated and fixed phosphorus from different soil compounds [77]. Similar to many other PGP *Bacillus* isolates, *Bacillus* sp. BioSol021 has also shown the ability of phosphate solubilisation, with a *PSI* of 2.03, which was higher compared to other phosphate-solubilizing strains isolated from alkaline wheat rhizosphere [101], but lower compared to strains isolated from the legume rhizosphere [100]. The presumed phosphate solubilisation mechanisms are usually related to pH regulation of the microenvironment by the production of organic acids arising from the phosphate-solubilising strains [77], whose mechanism of action is based on chelation of cations and competition with phosphates for adsorption sites in the soil [101], besides pH value regulation. For example, several organic acids, such as gluconic acid, malic acid, formic acid, succinic acid, lactic acid, malonic acid and citric acid were reported to be involved in effective phosphate solubilisation [102,103]. Production of organic acids is related to lowering the pH value of the cultivation medium, which was proven for the producing microorganism *Bacillus* sp. BioSol021 in our previous study [104]. Besides organic acid production, several more mechanisms are considered to be related to phosphate solubilisation, including production of phosphatases [105]. Inoculation of corn with phosphate-solubilizing bacteria was shown as a suitable path for the reduction in phosphate-based fertilizer application without yield loss [106]. The aforementioned reasons for poor phosphorus uptake by plants have resulted in a massive soil supply with phosphate fertilizers. However, around 80% of applied phosphorus becomes unavailable to plants due to complexation with Ca^2+^, Al^3+^ and Fe^3+^ ions available in the soil [107]. Therefore, the application of phosphate-solubilizing bacteria represents a convenient alternative for the reduction in dependency on phosphate fertilizers and the resulting negative environmental effects of the excessive application of inorganic fertilizers.

Ammonia production is a very important PGP trait, considering that the produced ammonia represents a direct PGP compound since it could be readily taken up and metabolized by plants [77]. *Bacillus* sp. BioSol021 cultivation broth has given a positive result in terms of ammonia production, hence being a candidate for direct plant growth promotion. Direct nitrogen supplementation of corn by microbially produced ammonia uptake has been shown to improve root and shoot elongation, as well as seedling fresh biomass [108]. Furthermore, accumulation of ammonia in the soil leads to soil alkalinization, thus contributing to unfavourable growth conditions for many phytopathogens resulting in their suppression [109]. Ammonia also acts as a metabolic inhibitor towards phytopathogens [110].

ACC deaminase hydrolyses ACC (1-aminocyclopropane-1-carboxylic acid) to α-ketobutyrate and ammonia, thus contributing to lower ethylene levels by ACC deamination, as ACC is a direct ethylene precursor [111]. Hence the bacteria that can use ACC as the sole nitrogen source are considered to express ACC deaminase activity, thus making a significant contribution to plant growth improvement by reducing ethylene synthesis under abiotic or biotic stress and reducing the consequent plant damage [77]. Lowering the ethylene level is especially important after seed germination in order to avoid ethylene inhibition of seedling root development, although ethylene is important for the start of germination as it breaks seed dormancy [111]. Facilitated root growth is a critical growth phase for seedling survival in the first days, hence ACC deaminase-mediated rooting of the seedling results in the development of mechanisms for stress alleviation even in later life phases, such as stress arising from drought or flooding, high salinity, heavy metals and phytopathogens [112]. Like many other *Bacillus* isolates, *Bacillus* sp. BioSol021 has shown a positive result in terms of growth in the medium containing ACC as the sole nitrogen source, hence confirming the ability of the producing strain to produce ACC deaminase and consequently contribute to plant stress alleviation, resulting in improved plant growth. Bacteria of the genus *Bacillus* as efficient ACC deaminase producers have shown to be suitable PGP agents for improved stress mitigation, growth and productivity of maize under different stress conditions, including drought and salinity stress [113,114,115].

### 2.4. Bacillus BioSol021 Immobilisation to Biochar and Maize Seed Germination

In order to investigate conditions for immobilisation of *Bacillus* sp. BioSol021 to soybean biochar, different independent variables related to biochar-microbial cell adhesion were assessed (biochar concentration in the cultivation broth and adhesion time) according to the experimental plan given in Table 5. The observed responses or dependent variables included different parameters related to the improvement of maize seed germination and seedling development in the initial growth phases (GP—germination percentage, RL—root length, SL—shoot length, FSM—fresh seedling mass, DSM—dry seedling mass, SVI—seedling vigour index). The overall aim was the maximisation of the following growth parameters: SVI, FSM and DSM, resulting in improved seed germination and seedling development, where the same weight coefficients were assigned to each of the examined dependent variables.

The results of the experiments included in the experimental plan (Table 5) in terms of the aforementioned dependent variables related to maize seed germination and seedling development are given in Table 6. Furthermore, observation of the separate experimental combinations of independent variables (immobilisation conditions) has provided the desirability function values for each experimental combination in order to maximise the aforementioned dependent variables, as described previously. 

The data presented in Table 6 show that almost every combination of the applied conditions for immobilisation of *Bacillus* sp. BioSol021 to biochar has resulted in a germination percentage of 100%, except experiment 7 (biochar concentration 1%, adhesion time 72 h) where the observed germination percentage was 95%, pointing out that the lowest biochar concentration and the longest duration of the immobilisation procedure from the examined ranges have resulted in the lowest germination percentage, as well as the lowest mean value of root length and fresh seedling mass together with the lowest value of seed vigour index. As presented in Table 6, immobilisation conditions including biochar concentration 5% and adhesion time 48 h (experiment 6) have resulted in the highest value of desirability function (0.9795). The value of desirability function is close to the next two combinations of immobilisation conditions (experiment 8–0.9440 and experiment 4–0.9470) which have shown a significant influence on maximisation of the investigated seedling development parameters. Observing the three aforementioned experiments, it could be observed that very high mean values of root length were achieved compared to other experimental runs, which also reflected in significantly higher values of seed vigour index, considering that quite high mean values of shoot length were achieved too. Experiment 6 with the highest desirability function value has also shown very high mean values of seedling fresh and dry mass. The maize root development is considered to be dependent on genetic determinants, but also responsive and adaptive to different environmental signals [116]. Embryogenesis is the stadium where the primary root and a number of seminal roots are formed, which become important for early seedling vigour after seed germination [117]. Among the investigated PGP traits, it was previously shown that IAA production and phosphate solubilisation contribute the most to root elongation and seedling dry mass increase in maize seed inoculated with *Bacillus* spp. [118]. *Bacillus*-produced IAA was also found responsible for maize stress alleviation and improved growth parameters under salinity and Al^3+^ stress [119,120]. *Bacillus* spp. were shown to promote the accumulation of amino acids and carbohydrates in maize, as one of the potential reasons for improved fresh and dry mass of maize seedlings [121]. On the other hand, the components of maize root exudate, including organic acids, amino acids and sugars, could contribute to improved *Bacillus* spp. biofilm formation resulting in improved maize root adherence and colonization [122], consequently leading to improved growth parameters. Similar results were observed by Zhang et al. [123], where glucose, citric acid and fumaric acid from maize root exudate stimulated growth and biofilm formation-related production of exopolysaccharide by *Bacillus amyloliquefaciens*, as well as induced the expression of PGP-related genes and genes responsible for secondary metabolism antibiotics synthesis. On the other hand, application of biochar derived from corn cob, rice straw and cocoa pod husk in the amount of 5 t/ha has significantly improved maize yield up to 2.5 t/ha [124]. Application of biochar resulted in increased soil pH value, water retention, as well as P and K availability, which provided alleviation of nutrient depletion stress and resulted in 363% higher content of maize aboveground biomass [125]. Improved water retention in the soil due to biochar addition was also one of the reasons for improved maize yield in the study by Benkova et al. [126]. The addition of biochar was also shown to promote biomass of maize stalk and root, as well as root morphology, leading to significantly higher potassium uptake, which resulted in 23.9% higher maize yield with a biochar application rate of 20 t/ha [127]. Application of coconut shells, coffee husks and maize cobs biochar resulted in maize yield increase of 1.0, 2.6, and 4.0 t/ha for biochar dosages of 1, 5, and 10 t/ha, respectively [128]. Furthermore, biochar addition also contributes to improved maize dry matter and nutrient (C, N, P, K) availability in the soil [129,130]. Biochar was also successfully applied as a soil amendment for the reduction in heavy metals (lead, cadmium, and chromium) content in maize plants and soil [131]. 

**Figure 9:**
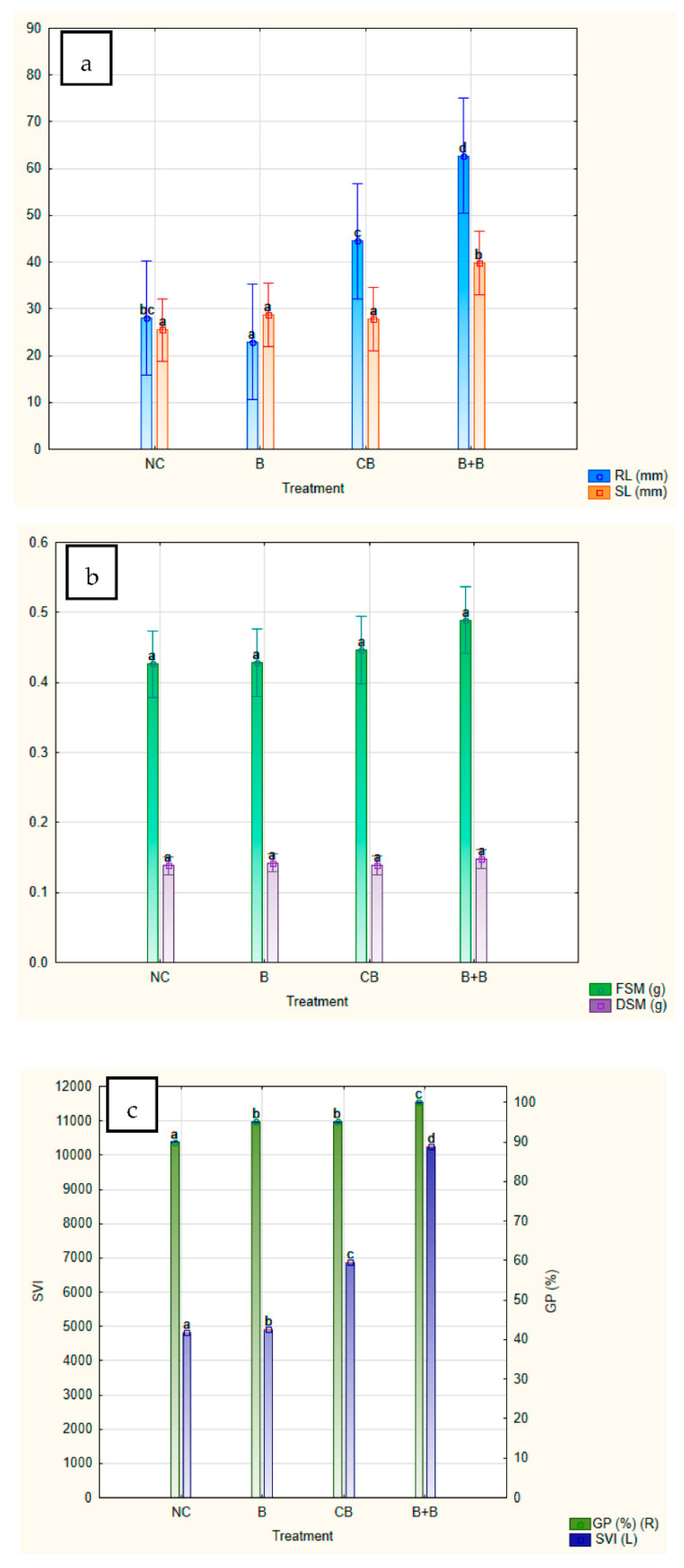
PGP parameters in terms of maize seed germination and seedling development: negative control (NC), biochar (B), cultivation broth of *Bacillus* sp. BioSol021 (CB), soil amendment based on biochar with immobilised cells of *Bacillus* sp. BioSol021 (B+B): (**a**)—root length (RL) and shoot length (SL), (**b**)—fresh seedling mass (FSM) and dry seedling mass (DSM), (**c**)—germination percentage (GP) and seedling vigour index (SVI).

In order to assess the synergistic effects of biochar with immobilised cells of *Bacillus* sp. BioSol021, the comparison was made between the *Bacillus*-loaded biochar from experiment 6 (B+B), where the highest value of desirability function in terms of PGP traits was achieved, and negative control (NC—no treatment), biochar addition as the soil amendment (B—0.02 g/g of substrate) and soil treatment using the *Bacillus* sp. BioSol021 cultivation broth (CB—0.2 mL/g of substrate). The comparative results in terms of seed germination and seedling growth parameters are given in Figure 9. 

Duncan’s multiple range test was used as a statistical tool to define homogenous groups of investigated PGP parameters at the same level of statistical significance (with a confidence level of 95%), taking into account the results for the following abovementioned samples: negative control (NC), biochar (B), cultivation broth of *Bacillus* sp. BioSol021 (CB) and soil amendment based on biochar with immobilised cells of *Bacillus* sp. BioSol021 (B+B). The differences in the manifested PGP activity are presented in Figure 8 with the displayed mean values and standard deviations of the observed PGP parameters. In the case of shoot length, the highest mean values were obtained for the soil amendment sample (B+B—39.80 ± 12.22 mm), with an approximately 1.5 fold increase compared to NC (25.45 ± 14.89 mm), but also compared to biochar (28.75 ± 14.02 mm) and *Bacillus* sp. BioSol021 cultivation broth (27.85 ± 18.60 mm) as separate components (Figure 9a). The presented results point out a very slight increase in shoot length when biochar and cultivation broth are applied as separate treatments, with a statistically significant difference in comparison to developed microbial biochar soil amendment. Similar results could be observed for root length obtained as a result of B+B application (62.70 ± 33.96 mm), whose value was almost three fold higher compared to NC (28.05 ± 22.36 mm) and B treatment (22.95 ± 14.12 mm), and significantly higher than CB treatment (44.45 ± 34.44). While the cultivation broth treatment has significantly improved maize root length, the addition of biochar resulted in lower mean root length compared to negative control. The presented results imply the undoubtedly positive effect of joint beneficial activity of biochar and *Bacillus* sp. BioSol021 cells on maize seedling development in the initial growth phases. The synergistic effect of those two components present in the soil amendment sample was also confirmed based on the results of FSM (Figure 9b), as well as GP and SVI (Figure 9c). None of the examined treatments besides microbial biochar soil amendment has resulted in GP of 100%. Higher GP and better seedling development parameters as a result of the B+B treatment have also resulted in a significantly higher SVI value (10250) compared to NC (4815), B (4911) and CB treatment (6868) (Figure 9c). The exception can be observed in the case of DM results, where almost similar values were obtained for each of the examined treatments (Figure 9b). 

**Figure 10:**
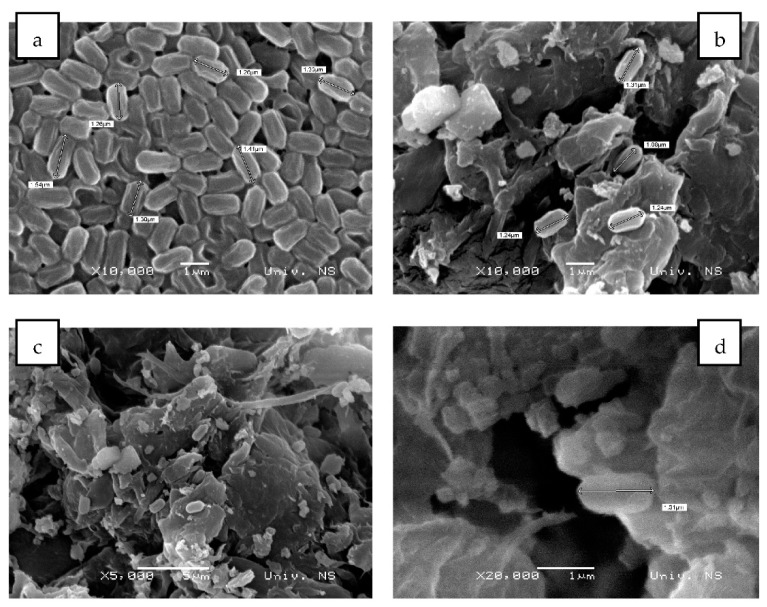
SEM images of free *Bacillus* sp. BioSol021 spores ((**a**)—×10,000 magnification) and biochar with immobilised spores ((**b**)—×10,000 magnification, (**c**)—×5000 magnification, (**d**)—×20,000 magnification).

Figure 10a shows SEM images of free spores of the producing microorganism *Bacillus* sp. BioSol021, while Figure 10b–d show biochar samples with immobilised cells using different magnifications. The samples used for SEM imaging were the *Bacillus* sp. BioSol021 cultivation broth (Figure 10a) and soybean biochar loaded with *Bacillus* sp. BioSol021 spores from experiment 6 (also used as B+B treatment), with the highest value of desirability function in terms of PGP traits (Figure 10b–d). The morphology of the spores of the producing microorganism has a typical appearance for representatives of the genus *Bacillus*. Rod cells, single or arranged in pairs, with an average diameter of 0.5 µm and length in the range of 1.2–1.6 µm [132]. The immobilised bacterial spores are attached to the structure of the porous material, located inside the pores and on the walls of the solid carrier, thus confirming the possibility to use biochar for successful cell immobilisation. The procedure of spore detachment from biochar and subsequent application of the plate count method resulted in a spore number of 8.34 log CFU/g. 

## 3. Materials and Methods

### 3.1. Production and Characterisation of Biochar

The biochar was obtained as a waste material from Sojaprotein Ltd., Bečej, Republic of Serbia, one of the biggest and most important NON GMO (genetically modified organisms) soy processing plants in Europe, with by far the biggest variety and quality of products, and annual capacity of 250,000 t. In order to produce the thermal energy required to power the Sojaprotein Ltd. facility, soybean plant stems and husks are used, and consequently biochar is generated as a waste material. During our investigations we used this waste after the thermal treatment sampled directly from the biochar storage. This material was sieved through ISO 3310-1:2016 standard set of sieves (sizes of 63, 125, 250, 315, 500, 710, and 1000 µm). Biochar pH value was measured in a water environment (10 g of ash/100 mL of demineralized water). Elemental analysis after sieving the biochar was performed by X-ray fluorescence spectroscopy (XRF) using the BRUKER mXRF ARTAX 200 system (Bruker Nano GmbH, Berlin, Germany), with Rh radiation source (25 kV, 1.5 mA) under a He atmosphere. SEM-EDS was performed on a JSM 6460LV (JEOL, Tokyo, Japan) electron microscope with Inca X-sight LN2 (Oxford Instruments, Abingdon, UK) energy-dispersive X-ray spectroscopy attachment, with direct observation of the samples without sputtering. The samples of *Bacillus* sp. BioSol021 spores present in the cultivation broth and biochar with immobilised spores were analysed.

### 3.2. Producing Microorganism, Cultivation Medium and Cultivation Conditions

In the present study *Bacillus* sp. BioSol021 was used as the producing microorganism, previously isolated from the rhizosphere of *Phaseolus vulgaris* [133]. The 16S rRNA sequencing and VITEK2 Compact System identification indicated the highest similarity of the selected microorganism was to the representatives of the operational group *Bacillus amyloliquefaciens* (GenBank sequence accession number ON569805) [134].

The optimal cultivation medium composition for *Bacillus* sp. BioSol021 cultivation was defined in a previous study and included (g/L): cellulose (5.0), (NH_4_)_2_SO_4_ (3.8), K_2_HPO_4_ (0.3) and MgSO_4_·7H_2_O (0.3) [135]. The inoculum preparation included transferring the loop of bacterial biomass to Erlenmeyer flasks containing nutrient broth (50 mL) (Himedia, Thane (West), Maharashtra, India), and cultivation on a rotary shaker at 28 °C and 170 rpm, under spontaneous aeration for 24 h. The same experimental conditions were applied in the second step of inoculum preparation, when the liquid culture was transferred to the Erlenmeyer flasks of higher volume (500 mL) containing the same commercial medium (150 mL), and cultivated for another 24 h. Inoculation of cultivation media for biosynthesis (10 L) was performed by adding 10% (*v/v*) of the prepared inoculum (1 L). The cultivation was carried out in a 16 L-laboratory bioreactor (EDF—15.4_1, A/S Bio-tehniskais centre, Riga, Latvia), at the agitation rate of 300 rpm and aeration rate of 1.5 vvm—volume of sterile air/(volume of the medium·min). The initial pH value of the medium was set to 7.0 ± 0.2 prior to sterilization by autoclaving, the bioprocess temperature was 28 °C, and the cultivation time 96 h.

### 3.3. Characterisation of Cultivation Broth—Biomass, Residual Nutrients, pH Value

The number of viable cells of *Bacillus* sp. BioSol021 in the cultivation broth sample was determined by the plate count method and given as CFU/mL (CFU—colony forming units). The carbon and nitrogen source content were determined using standard analytical methods [136,137]. Cultivation broth pH value was recorded at the end of the cultivation using the pH electrode (EasyFerm Bio HB K8 425, Hamilton, Bonaduz, Switzerland).

### 3.4. Characterisation of Cultivation Broth—Plant Growth Promoting and Biocontrol Traits

The PGP/biocontrol potential of the produced cultivation broth using *Bacillus* sp. BioSol021 was estimated by performing the testing of the key parameters described below. 

#### 3.4.1. CPC-BTB Method for Surfactin Quantification

The surfactin concentration in the *Bacillus* sp. BioSol021 cell-free supernatant was measured by employing the CPC-BTB (cetylpyridinium chloride—bromothymol blue) method [53]. The cell-free supernatant was obtained by centrifugation of the cultivation broth sample at 12,000 rpm for 10 min (Z 326 K, Hermle LaborTechnik GmbH, Wehingen, Germany). Bromothymol blue (BTB) and the mediator cetylpyridinium chloride form a green coloured complex. When surfactin is added, it forms a colourless complex with the CPC, releasing BTB molecules to the medium and generating a colour shift that can be detected spectrophotometrically. The quantification was carried out by mixing 300 μL of the supernatant sample and 2.4 mL of the CPC-BTB reagent and the mixture was kept at 25 °C for 5 min. Finally, absorbance at 600 nm was measured (UV 1800, Shimadzu, Kyoto, Japan) and the surfactin concentration was calculated using the standard curve [138].

#### 3.4.2. PGP Traits Screening

The colorimetric determination of IAA content was performed using the method described by Syed-Ab-Rahman et al. [139], with slight modifications. Briefly, 1 mL of the *Bacillus* sp. BioSol021 cultivation broth cell-free supernatant was mixed with 2 mL of Salkowski reagent (1.2% (*w/v*) FeCl_3_ in 7.9 M H_2_SO_4_) and incubated in the dark at room temperature for 30 min. The appearance of a pink colour indicates the ability of the producing microorganism to produce IAA. After 30 min, the spectrophotometric measurement was performed at a wavelength of 535 nm (UV 1800, Shimadzu, Kyoto, Japan). Distilled water was used as a blank sample, and the calibration curve was prepared using the IAA standard (Sigma-Aldrich, MO, USA).

The proteolytic activity of the obtained cultivation broth was assayed using modified skim milk agar [140]. The medium composition included (g/L): skim milk 28, tryptone 5, yeast extract 2.5, glucose 1, agar 15. The pH value was adjusted to 7.0 ± 0.2 and the medium was sterilized by autoclaving (121 °C, 2.1 bar, 20 min). After inoculation of the prepared skim milk agar plates using 1 µL of the cultivation broth, incubation was carried out for 120 h at 28 °C. The evaluation of the enzymatic activity was performed based on the appearance of the biomass growth and the clear zone around it. The extracellular enzymatic activity index (*EAI*) was calculated according to the following equation for protease and the following enzymes assayed:

(1) $$EAI=\frac{diameter\;of\;biomass\;growth+diameter\;of\;halo\;zone}{diameter\;of\;biomass\;growth}.$$

The cellulase and xylanase activity of the producing microorganism *Bacillus* sp. BioSol021 cultivation broth was assayed using the media with the following composition (g/L): CMC or xylan 5, glucose 1, yeast extract 0.5, KCl 1, K_2_HPO_4_ 1, NaNO_3_ 1, MgSO_4_·7H_2_O 0.5, agar 17 [141]. Inoculation and incubation conditions were the same as previously described in 3.3.1. For a better visualization of the clear zones around microbial growth indicating cellulase/xylanase activity, the media after the incubation were poured over, using the Congo red solution (0.5% (*w/v*), 15 min) and afterwards the NaCl solution (1 M, 15 min). The measurement of the growth and halo zones was performed 10 min after the removal of the NaCl solution.

The pectinase activity of the producing microorganism *Bacillus* sp. BioSol021 cultivation broth was assayed using medium with composition (g/L): pectin 10, yeast extract 0.5, KCl 1, K_2_HPO_4_ 1, NaNO_3_ 1, MgSO_4_·7H_2_O 0.5, agar 20 [142]. Inoculation and incubation conditions were the same as previously described in 3.3.1. Visualization of the halo zones was performed after pouring over Gram’s iodine solution (5 min) and washing it with distilled water.

The phosphate solubilization test was performed using Pikovskaya’s medium (Himedia, Thane (West), Maharashtra, India). After medium inoculation using 1 µL of the *Bacillus* sp. BioSol021 cultivation broth, the incubation was performed under the previously described conditions for enzymatic activity screening. After 5-day incubation, growth and halo zone diameters were measured, while the phosphate solubilisation index (*PSI*) was calculated according to the following formula [143]:

(2) $$PSI=\frac{diameter\;of\;biomass\;growth+diameter\;of\;halo\;zone}{diameter\;of\;biomass\;growth}.$$

Cultivation broth sample (10 mL) was mixed with Nessler’s reagent (0.5 mL) and incubated at room temperature for 5 min. Appearance of yellow or brownish colour indicates a positive test for ammonia production by the producing microorganism *Bacillus* sp. BioSol021.

In order to check ACC deaminase activity, cultivation broth (1 µL) was inoculated on minimal DF (Dworkin and Foster) salts medium containing 3 mM ACC as a sole nitrogen source [111]. Incubation was performed as previously described, while the appearance of the *Bacillus* sp. BioSol021 colony growth on the medium was considered a positive result in terms of ACC deaminase production.

### 3.5. Immobilisation of Producing Microorganism on Biochar

The immobilisation procedure included mixing the cultivation broth of producing microorganism *Bacillus* sp. BioSol021 produced in a 16 L laboratory bioreactor and biochar in a proportion defined by the experimental plan (Table 5). Incubation was carried out on a laboratory shaker (KS 4000i control, IKA^®^ Werke, Staufen, Germany) with external stirring at 150 rpm at 28 °C, in Erlenmeyer flasks containing 30 mL of cultivation broth and biochar in the amount given in the experimental plan. The experimental plan included a full experimental design with two independent variables, the mixing time and amount of biochar, varied at three levels. After the incubation time, biochar with immobilised cells of the producing microorganism was separated from the rest of the cultivation broth by filtration (Whatman #1, Cytiva, Marlborough, MA, USA) in a gravity field and dried at 40 °C for 24 h. In order to determine the number of *Bacillus* sp. BioSol021 cells bound to biochar, the next step involved resuspending 0.1 g of biochar with the attached cells in 10 mL of sterile saline solution and incubation for 24 h on a laboratory shaker (KS 4000i control, IKA^®^ Werke, Staufen, Germany) with intensive external mixing at 200 rpm.

#### SEM Imaging of the Free and Biochar-Bound *Bacillus* sp. BioSol021 Cells 

Scanning electron microscopy (SEM) was used for imaging the producing microorganism’s cells, free and attached to biochar particles as a solid carrier. The SEM analysis was performed using a sample of biochar with immobilised cells of the producing microorganism obtained in experimental set No. 6, by mixing solid carrier in a concentration of 5% (*w*/*v*) and the *Bacillus* sp. BioSol021 cultivation broth for 48 h. Imaging was performed using a scanning microscope (JSM 6460 LV, JEOL, Tokyo, Japan) with an EDS detector (The Energy Dispersive X-Ray Detector—INCA EDS, Oxford Instruments, Abingdon, UK) at a voltage of 20 kV and a working distance of 13 mm. 

### 3.6. Seed Germination Activity Assay

Maize seeds *(Zea mays* subsp. *mays*) were used for the plate seed germination assay, using the samples from the experimental design given in Table 5. The surfaces of the maize seeds were sterilized using chlorine bleach solution (6% (*v/v*), 1 min) and thoroughly washed using sterile distilled water for 5 min. After drying, a total number of 20 seeds were placed in a Petri dish (90 mm) containing the substrate (5 g, Seedlingsubstrat, Klasman-Deilmann, Geeste, Germany) mixed with biochar samples after *Bacillus* sp. BioSol021 cells attachment (0.1 g) or biochar sample without bound *Bacillus* sp. BioSol021 cells (0.1 g). Seeds in one of the Petri dishes were treated using 1 mL of the *Bacillus* sp. BioSol021 cultivation broth. The substrate was provided with 10 mL of tap water, while the negative control represented non-treated maize seeds. The seeds’ incubation was performed in an incubator at 25 °C for 7 days, followed by enumeration of germinated seeds, root and shoot length measurement, as well as the seedlings’ fresh and dry mass measurement (after drying at 105 °C until reaching the constant mass). The seedling vigour index (*SVI*) was calculated using the following formula [109,144]:

(3) SVI=GP·TSL

where *GP* is germination percent (%) and *TSL* is total seedling length (mm).

### 3.7. Experimental Data Analysis

The statistical data analysis was performed using Duncan’s multiple range test in the Statistica 13.2 software (Dell, TX, USA), with all tests performed at the significance level of 0.05 (95%). Desirability function values were determined using Python Pandas and Scikit-Learn libraries v. 1.3.1, aiming for the maximisation of the following parameters: FSM, DSM and SVI. Desirability functions were calculated by assigning the same weight coefficients to each of the aforementioned parameters.

## 4. Conclusions

This study aimed at development of a novel microbial soil amendment based on PGP and biocontrol strain *Bacillus* sp. BioSol021 and soybean biochar. The results of biochar physicochemical analyses have confirmed its suitability for agricultural applications for particles smaller than 1000 μm. On the other hand, *Bacillus* sp. BioSol021 cultivation broth has shown suitable PGP traits. Investigation of different conditions for *Bacillus* sp. BioSol021 immobilisation to biochar was performed by simultaneously monitoring seed germination activity and PGP traits of microbial biochar soil amendment obtained under different conditions of bacterial immobilisation. The desirability function method, aimed at maximisation of SVI, FSM and DSM, has shown that the most suitable conditions were biochar concentration of 5% and adhesion time of 48 h. Soil amendment produced in the aforementioned way had a significantly higher influence on maize seed germination and initial seedling development compared to separate treatments including only biochar or cultivation broth. Hence, the synergistic effect of the beneficial *Bacillus* strain and soybean biochar was proven in this study, opening a new chapter of possibilities to explore different types of biochar produced from various raw materials to be used as solid carriers for beneficial PGP and biocontrol microbial strains. The next steps will include greenhouse and field testing of the developed soil amendment in order to evaluate its effectiveness in PGP in a wider range of economically significant crops.

## Figures and Tables

**Table 1:** Results of EDS analysis for biochar particle fraction in the size range 63–125 μm.

Spectrum	C (%)	O (%)	Mg (%)	Al (%)	Si (%)	S (%)	Cl (%)	K (%)	Ca (%)	Fe (%)	Sb (%)	I (%)	Total
Spectrum 1	0.00	42.62	7.20	0.00	23.20	0.00	0.00	14.29	10.05	0.00	1.90	0.73	100.00
Spectrum 2	10.43	49.30	4.57	0.45	1.97	0.42	0.29	2.07	30.21	0.29	0.00	0.00	100.00

**Table 2:** Results of EDS analysis of biochar particle fraction in size range 125–250 μm.

Spectrum	C (%)	O (%)	Na (%)	Mg (%)	Al (%)	Si (%)	P (%)	S (%)	Cl (%)	K (%)	Ca (%)	Fe (%)	Sb (%)	Total
Spectrum 1	16.48	21.68	0.00	2.04	0.25	1.36	1.34	7.45	7.73	30.98	9.97	0.72	0.00	100.00
Spectrum 2	88.37	49.30	0.00	0.30	0.00	0.00	0.90	0.00	0.08	1.22	0.43	0.00	0.00	100.00
Spectrum 3	0.00	53.21	0.55	2.59	0.38	22.25	0.00	0.00	0.99	15.04	3.62	0.52	0.58	100.00

**Table 3:** Composition of the *Bacillus* sp. BioSol021 cultivation broth.

Parameter	Value
Residual cellulose content (g/L)	2.8
Residual total nitrogen content (g/L)	0.27
Biomass content (log(CFU/mL))	8.47
pH value	6.3

**Table 4:** The results of PGP/biocontrol traits’ screening for cultivation broth of *Bacillus* sp. BioSol021.

Parameter	Concentration (mg/L)	Halo Zone Diameter (mm)	*EAI* ^1^	*PSI* ^2^	Positive/ Negative Test
Surfactin production	1475	/	/	/	/
IAA production	15	/	/	/	/
Cellulase activity	/	54.50 ± 0.50	2.71	/	/
Xylanase activity	/	32.00 ± 0.50	2.85	/	/
Pectinase activity	/	21.00 ± 1.00	3.58	/	/
Protease activity	/	31.00 ± 0.00	1.55	/	/
Phosphate solubilisation	/	11.50 ± 0.50	/	2.03	/
Ammonia production	/	/	/	/	+
ACC deaminase production	/	/	/	/	+

^1^*EAI*—enzyme activity index, ^2^*PSI*—phosphate solubilization index.

**Table 5:** Experimental plan for the immobilisation of the *Bacillus* sp. BioSol021 cells on the biochar.

Number of Experiment	Incubation Time (h)	Biochar Amount (%, *w/v*)
1	24	1
2	24	3
3	24	5
4	48	1
5	48	3
6	48	5
7	72	1
8	72	3
9	72	5

**Table 6:** Desirability function values for varied experimental conditions in terms of *Bacillus* sp. BioSol021 immobilisation on biochar aimed at maximisation of the presented maize seed germination and seedling development parameters considering experimental data without outliers.

Experiment	GP (%)	RL (mm)	SL (mm)	FSM (g)	DSM (g)	SVI	Desirability (%)
2	100 ^c^	34.20 ± 25.46 ^a^	34.20 ± 12.92 ^bc^	0.43 ± 0.09 ^a^	0.12 ± 0.03 ^a^	6840 ^d^	0.7702
7	95 ^b^	32.00 ± 22.11 ^a^	33.85 ± 14.09 ^abc^	0.42 ± 0.07 ^a^	0.14 ± 0.03 ^abc^	6255 ^b^	0.7709
5	100 ^c^	42.70 ± 33.99 ^abc^	32.10 ± 13.10 ^ab^	0.43 ± 0.09 ^a^	0.13 ± 0.03 ^abc^	7480 ^f^	0.8131
1	100 ^c^	33.95 ± 18.70 ^a^	33.70 ± 11.95 ^abc^	0.45 ± 0.09 ^a^	0.15 ± 0.02 ^c^	6765 ^c^	0.8357
9	100 ^c^	37.30 ± 19.71 ^ab^	31.35 ± 9.21 ^ab^	0.45 ± 0.09 ^a^	0.15 ± 0.03 ^c^	6865 ^e^	0.8410
3	100 ^c^	57.10 ± 26.24 ^cde^	42.00 ± 12.44 ^c^	0.45 ± 0.08 ^a^	0.13 ± 0.03 ^ab^	9910 ^h^	0.9009
8	100 ^c^	71.60 ± 23.29 ^e^	34.25 ± 9.80 ^bc^	0.46 ± 0.08 ^a^	0.14 ± 0.03 ^abc^	10585 ^j^	0.9440
4	100 ^c^	53.00 ± 31.18 ^bcd^	39.00 ± 11.31 ^bc^	0.49 ± 0.10 ^a^	0.15 ± 0.04 ^c^	9200 ^g^	0.9470
6	100 ^c^	62.70 ± 33.96 ^de^	39.80 ± 12.22 ^bc^	0.49 ± 0.11 ^a^	0.15 ± 0.02 ^bc^	10250 ^i^	0.9795
NC	90 ^a^	28.05 ± 22.36 ^a^	25.45 ± 14.89 ^a^	0.426 ± 0.10 ^a^	0.138 ± 0.025 ^abc^	4815 ^a^	-

GP (%)—germination percentage, RL (mm)—root length, SL (mm)—shoot length, FSM (g)—fresh seedling mass, DSM (g)—dry seedling mass, SVI—seed vigour index, NC—negative control. Superscript letters designate different levels of statistical significance (with *p*-values over 0.05) according to Duncan’s multiple range test.

## Data Availability

Not applicable.
